# Feasibility and outcomes of living-donor liver transplantation utilizing the right hemi-liver graft with portal vein anatomical variations

**DOI:** 10.1007/s00423-023-03115-9

**Published:** 2023-10-04

**Authors:** Ahmed Shehta, Mohamed Elshobari, Tarek Salah, Ahmad M. Sultan, Amr Yasen, Usama Shiha, Mohamed El-Saadany, Ahmed Monier, Rami Said, Mohamed S. Habl, Reham Adly, Basma Abd Elmoaem El Ged, Rasha Karam, Reem Khaled, Hassan Magdy Abd El Razek, Ehab E. Abdel-Khalek, Mohamed Abdel Wahab

**Affiliations:** 1 Gastrointestinal Surgery Center, Department of Surgery, Faculty of Medicine, Mansoura University, Gehan Street, Mansoura, Postal code: 35516 Egypt; 2 Liver Transplantation Unit, Gastrointestinal Surgery Center, Department of Anesthesia, Faculty of Medicine, Mansoura University, Mansoura, Egypt; 3 Liver Transplantation Unit, Gastrointestinal Surgery Center, Department of Radiology, Faculty of Medicine, Mansoura University, Mansoura, Egypt; 4 Liver Transplantation Unit, Gastrointestinal Surgery Center, Department of Hepatology, Faculty of Medicine, Mansoura University, Mansoura, Egypt

**Keywords:** Living-donor liver transplantation, Portal vein, Anatomical variations, Long-term outcomes

## Abstract

**Purpose:**

Portal vein (PV) reconstruction is a key factor for successful living-donor liver transplantation (LDLT). Anatomical variations of right PV (RPV) are encountered among potential donors.

**Methods:**

To evaluate a single center experience of reconstruction techniques for the right hemi-liver grafts with PV variations during the period between May 2004 and 2022.

**Results:**

A total of 915 recipients underwent LDLT, among them 52 (5.8%) had RPV anatomical variations. Type II PV was found in 7 cases (13.5%), which were reconstructed by direct venoplasty. Type III PV was found in 27 cases (51.9%). They were reconstructed by direct venoplasty in 2 cases (3.8%), Y graft interposition in 2 cases (3.8%), and in situ double PV anastomoses in 23 cases (44.2%). Type IV PV was found in 18 cases (34.6%) and was reconstructed by Y graft interposition in 9 cases (17.3%), and in situ double PV anastomoses in 9 cases (17.3%).

Early right posterior PV stenosis occurred in 2 recipients (3.8%). Early PV thrombosis occurred in 3 recipients (5.8%). The median follow-up duration was 54.5 months (4 – 185). The 1-, 3-, and 5-years survival rates were 91.9%, 86%, and 81.2%, respectively. Late PV stenosis occurred in 2 recipients (3.8%) and was managed conservatively.

**Conclusion:**

Utilization of potential living donors with RPV anatomic variations may help to expand the donor pool. We found that direct venoplasty and in situ dual PV anastomoses techniques were safe, feasible, and associated with successful outcomes.

## Introduction

Liver transplantation is widely accepted as the only effective treatment for patients with end stage liver disease and selected hepatic malignancies [1]. In Egypt, deceased donor liver transplantation (DDLT) program is still awaited because of social and ethical aspects, making living-donor liver transplantation (LDLT) the only hope for those patients [2, 3].

The first adult LDLT was performed in 1994 [4]; afterwards, the use of the right hemi-liver graft has become popular in different liver transplantation centers. This is attributed to the larger volume of liver tissue provided by the right hemi-liver graft that is appropriate for adult liver transplant recipients [5]. On the other hand, the right hemi-liver graft is associated with high incidence of vascular tree and biliary tract anatomical variations, which add more surgical challenges and difficulties [6, 7].

Portal vein (PV) reconstruction is a key factor to achieve a successful LDLT surgery. Anatomical variations of the right PV (RPV) are commonly encountered among potential donors. The incidence of portal venous variations had been heterogenous between different series ranging between 10% and 35% [5, 8, 9]. Different surgical techniques have been described to overcome these anatomical variations like back wall venoplasty, autologous Y-graft interposition, in situ dual PV anastomoses, and Malatya approach utilizing autologous saphenous vein graft [10–13]. However, the most ideal surgical technique for reconstruction of RPV anatomical variations remains controversial.

The current study was conducted to estimate the incidence of RPV anatomical variations among liver donors and evaluate different reconstruction techniques utilized in portal flow reconstruction for the right hemi-liver graft with PV anatomical variations. To the best of our knowledge, this is the first study from Egyptian centers addressing the feasibility and the outcomes of adult LDLT utilizing right hemi-liver grafts with PV anatomical variations.

## Patients and methods

We retrospectively reviewed the data of all recipients who underwent LDLT at Liver Transplantation Unit, Gastrointestinal Surgery Center, Mansoura University during the period between May 2004 and May 2022. Recipients who underwent LDLT utilizing right hemi-liver grafts from living donors with RPV anatomical variations were identified and included in the current study. Recipients’ data were retrieved from a prospectively maintained database for all recipients undergoing LDLT. An informed consent was obtained from each recipient and donor. The study was approved by the Institutional Review Board and Local Ethical Committee at the Faculty of Medicine, Mansoura University, Egypt with reference number (R.23.01.2003).

### Preoperative preparation

Preoperative evaluation protocol had been described previously [2, 14]. In summary, preoperative evaluation of potential recipients included 4 phases: Phase I included detailed laboratory and radiological evaluation, and anesthetic consultation. Phase II included detailed cardiopulmonary and neuro-psychiatric evaluation. Phase III included gastrointestinal endoscopic evaluation. Phase IV included routine general consultations to exclude possible hidden septic foci.

For donors, evaluation of PV anatomical variation was dependent of computed tomographic portography. Classification of PV anatomical variations was based upon Cheng classification where: Type I, classical anatomical division of the main PV; Type II, trifurcation of the main PV; Type III, the right posterior PV arises as the first branch of the main PV; Type IV, the right anterior PV arises from the left PV [15].

### Operative technique

The operative technique had been described before [2, 14]. A standard right hemi-liver graft was commonly used for adult LDLT. The liver graft was implanted into the hepatic fossa. Reconstruction of the hepatic venous outflow was done first, followed by portal, arterial, and biliary reconstruction.

**Fig. 1:**
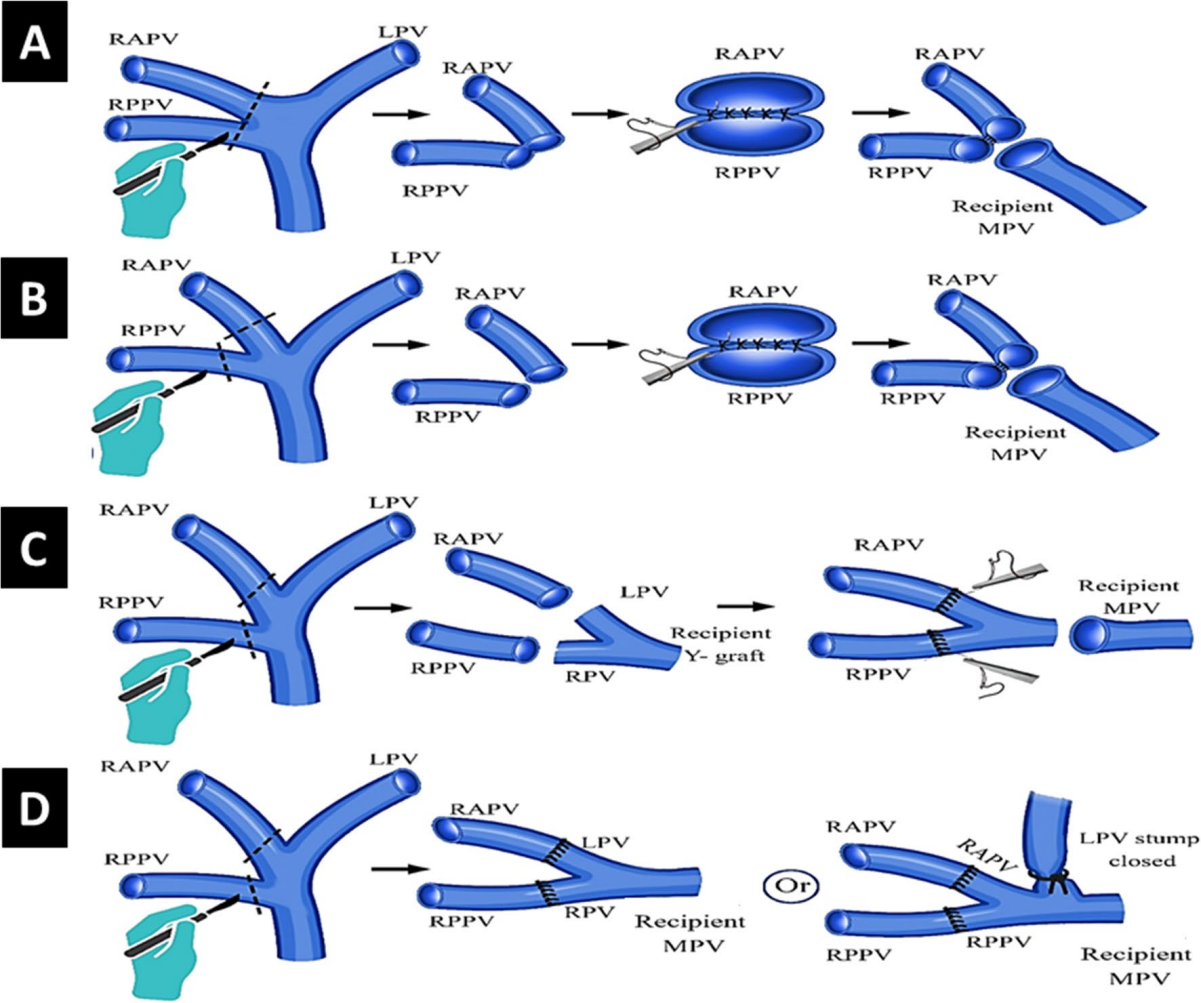
Different reconstruction techniques of portal vein variations. **A**, **B**: Direct venoplasty technique. **C**: Autologous Y-graft interposition technique utilizing the recipient’s portal vein bifurcation. **D**: In situ direct dual PV anastomoses technique. RPV right portal vein, RAPV right anterior portal vein, RPPV right posterior portal vein, LPV left portal vein, MPV main portal vein

**Fig. 2:**
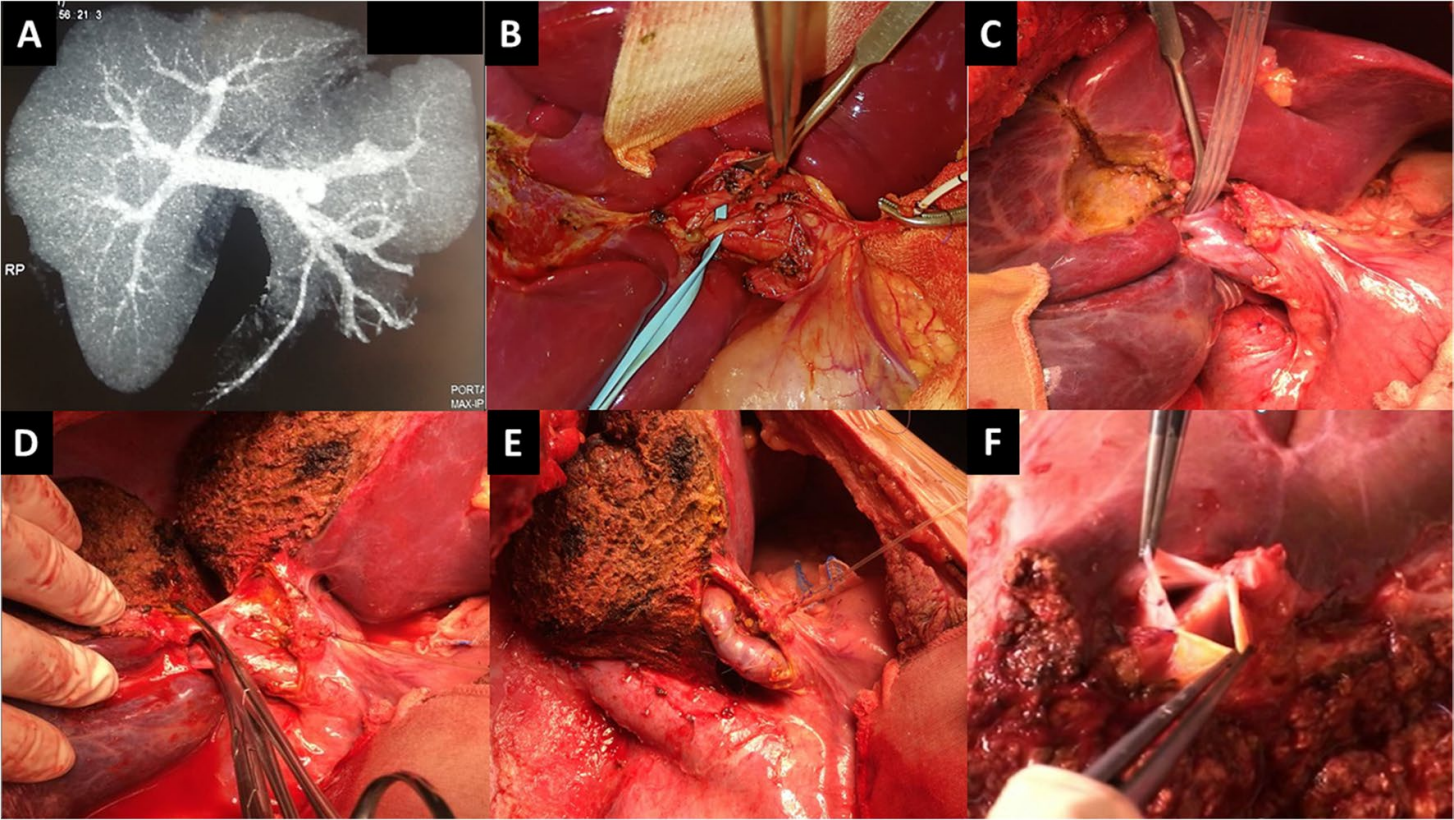
Direct venoplasty technique for type II portal vein. **A**: Donor CT portography showing trifurcated portal vein. **B**: Donor hilar dissection and identification of two separate right portal vein branches. **C**: Hanging maneuver and identification of the transection plane. **D**: Clamping of both branches of right portal vein in antero-posterior direction. **E**: Operative view after graft extraction and closure of right portal vein orifices. **F**: Creation of a common orifice of portal vein branches by direct venoplasty technique at the back table

For liver grafts with RPV anatomical variations, different techniques were utilized for PV reconstruction. If the two RPV branches were close to each other, as in type II PV, a common orifice was created by direct venoplasty utilizing polypropylene 6/0 sutures at the back table to be anastomosed as a single orifice to recipient PV. In some donors, the RPV was divided at the level of the trifurcation leaving a small bridge between the two PV branches. The intervening bridge was widened by venoplasty utilizing polypropylene 6/0 sutures at the back table. Similarly, a single PV anastomosis is created with the recipient PV (Figs. 1A, B and 2).

If the two RPV branches were far from each other, as in type III and IV PV, we utilized either autologous Y-graft interposition technique or in situ direct dual PV anastomoses technique. For autologous Y-graft interposition technique, the recipient’s PV bifurcation was excised with the proximal main PV trunk and used as a Y-graft. The recipient right and left portal branches were anastomosed to the two separate graft RPV branches utilizing polypropylene 6/0 sutures at the back table. The distal end of the autologous Y-graft was then anastomosed to the remnant recipient main PV as a single anastomosis in the recipient (Fig. 1C).

**Fig. 3:**
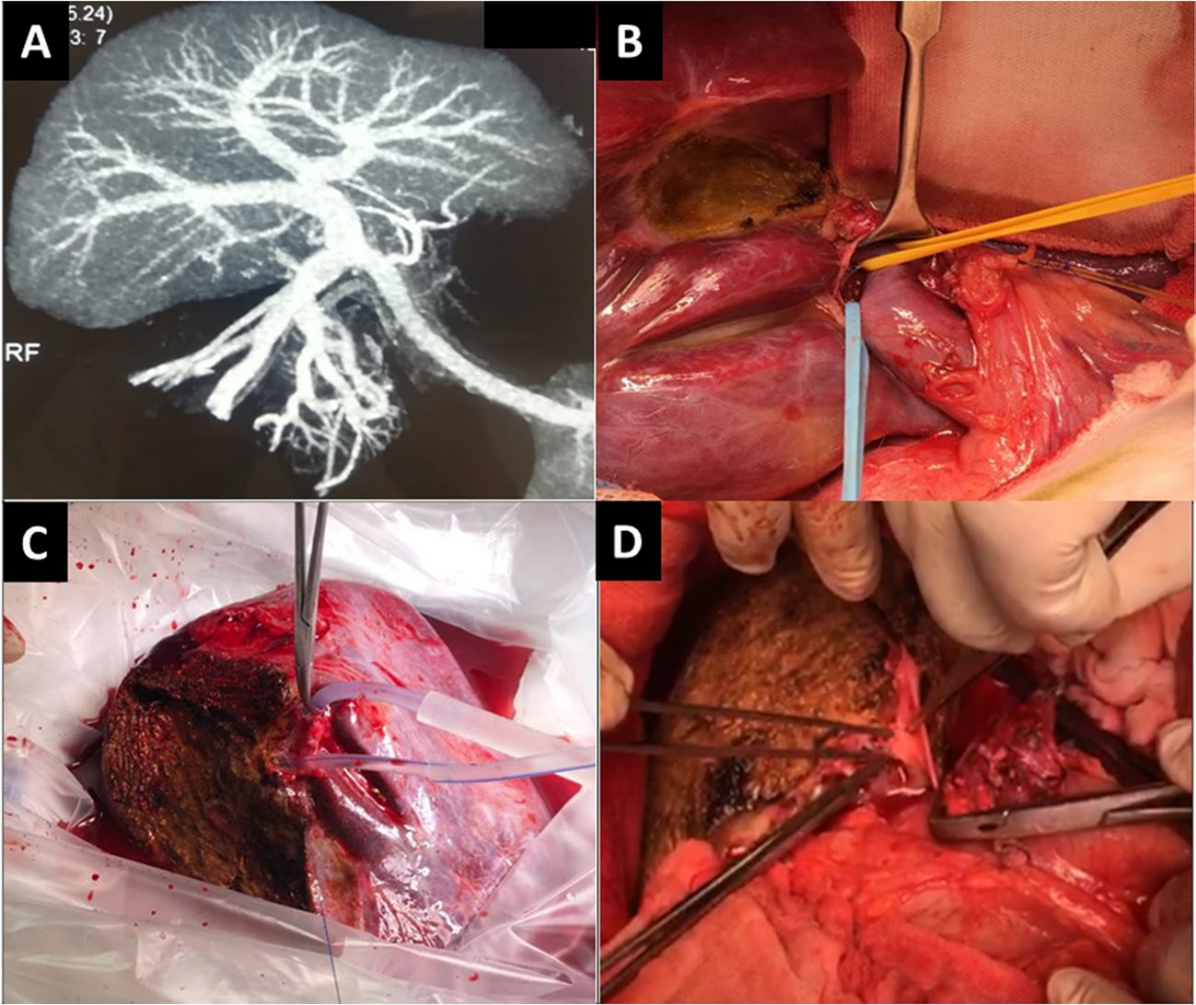
In situ direct dual portal vein anastomoses technique for type III portal vein. **A**: Donor CT portography showing double right portal vein branches. **B**: Donor Hilar dissection and identification of two separate right portal vein branches. **C**: During bench surgery with infusion of the two widely separated portal vein branches. **D**: Anastomosis of the two separate right portal vein branches to the recipient right and left portal vein branches

**Fig. 4:**
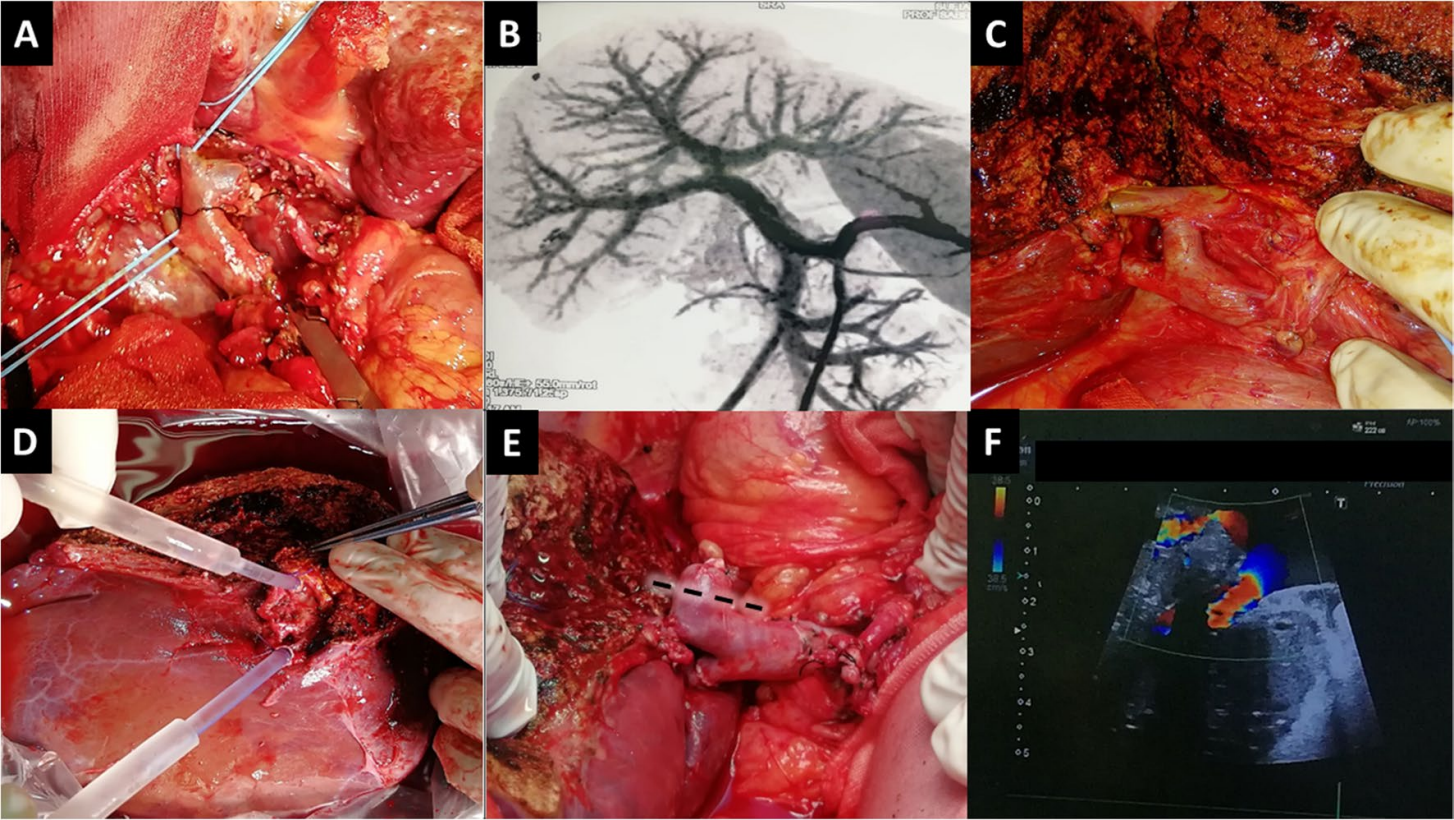
In situ direct dual portal vein anastomoses technique for type IV portal vein. **A**: Recipient hilar dissection showing type IV portal vein similar to the donor. **B**: Donor CT portography showing double right portal vein branches. **C**: Donor operative view before division of right portal vein branches. **D**: During bench surgery with infusion of the two widely separated portal vein branches. **E**: Anastomosis of the two separate right portal vein branches to the recipient right anterior and posterior portal vein branches. (left portal vein stump is shortened afterwards, dotted line) **F**: Intraoperative doppler ultrasound after completion of portal vein reconstruction

For in situ direct dual PV anastomoses technique, the two separate graft RPV branches were anastomosed each to the recipient right and left PV branches utilizing polypropylene 6/0 sutures. This required an acceptable angulation of the recipient main PV axis (Figs. 1D and 3). In few recipients with type III or IV PV, the two separate graft RPV branches were anastomosed each to the corresponding recipient right anterior and posterior PV branches with closure of the recipient left PV stump utilizing polypropylene 6/0 sutures (Figs. 1D and 4).

Intraoperative Doppler ultrasound was used to confirm the patency of all vascular anastomoses. A portal vein flow around 100 – 300 ml/min/100 g liver graft was considered adequate. After ensuring adequate hemostasis and biliostasis, abdominal drains were placed around the graft and near the raw surface.

### Postoperative care and follow-up

After surgery, all cases were transferred to the intensive care unit (ICU) for monitoring. Patients underwent detailed laboratory evaluation twice daily during the ICU stay. Oral intake and ambulation were allowed once bowel sounds were restored. Patients were transferred to the ward on the fifth postoperative day, depending on clinical improvement. Doppler ultrasound (US) played the main role in the follow-up of the recipients. Our postoperative protocol included doppler US examinations once daily during the first week, day after other during the second and third weeks and before hospital discharge, and then once weekly during the following 2 months. Further radiological workup was done upon suspicion of any postoperative complications.

After discharge, patients were followed up regularly on outpatient visits. Follow-up visit included detailed history taking, clinical examination, detailed laboratory evaluation including trough level of immunosuppression drugs, and Doppler US evaluation of hepatic vasculature.

### Definitions

Postoperative morbidity is defined as any adverse event occurring during the first 90 postoperative days and is graded according to the Clavien-Dindo classification. Major complications are defined as class 3 or higher [16]. Postoperative liver dysfunction, biliary fistula, and hemorrhage are defined according to the ISGLS definitions [17–19]. Early postoperative mortality was defined as mortality occurring during the first 90 postoperative days and was excluded from further survival analysis. Late morbidities were defined as morbidities occurring after the first 90 postoperative days. Late mortality was defined as mortality occurring after the first 90 postoperative days. Overall survival (OS) was calculated from the day of surgery to the day of confirmed death or the last follow-up visit.

### Data analysis

Shapiro–Wilk test was used to assess the normality of the data. Categorical variables were expressed as number and percentage, and continuous variables were expressed as median and range. Survival analysis was performed by Kaplan–Meier analysis. Statistical analysis was performed using the SPSS 22 software (IBM, Chicago, IL, USA). A *p* value less than 0.05 was considered statistically significant.

## Results

During the period between May 2004 and May 2022, 915 recipients underwent LDLT at Liver Transplantation Unit, Gastrointestinal Surgery Center, Mansoura University. Fifty-two recipients (5.8%) underwent LDLT utilizing right hemi-liver grafts from living donors with RPV anatomical variations and were included in the current study.

### Preoperative demographic data

The preoperative demographic data of the study cases were summarized in Table 1. The most common indications for liver transplantation were hepatitis C virus related liver cirrhosis in 32 recipients (61.5%), followed by hepatocellular carcinoma (HCC) in 14 recipients (26.9%).

**Table 1:** Preoperative demographic data of the study cases

Variables	Number of patients (*N* = 52)
Age (years)	50 (18 – 67)*
Sex	
Male	44 (84.6%)
Female	8 (15.4%)
Body mass index (kg/m^2^)	29.1 (18.2 – 36.1)*
Presentation	
Encephalopathy	12 (23.1%)
Jaundice	32 (61.5%)
Ascites	17 (32.7%)
Bleeding	13 (25%)
Edema of lower limbs	17 (32.7%)
Weight loss	3 (5.8%)
Indication of liver transplantation	
Hepatitis C virus related cirrhosis	32 (61.5%)
Hepatocellular carcinoma	14 (26.9%)
Within Milan Criteria	12 (23.1%)
Within UCSF Criteria	2 (3.8%)
Autoimmune hepatitis	4 (7.7%)
Budd-Chiari syndrome	1 (1.9%)
Cryptogenic cirrhosis	1 (1.9%)
Preoperative laboratory	
Albumin (g/dl)	3 (2.2 – 4.5)*
Bilirubin (mg/dl)	2.5 (0.3 – 20)*
International normalized ratio	1.45 (1 – 3.1)*
Creatinine (mg/dl)	0.8 (0.5 – 1.6)*
Alpha feto-protein (ng/ml)	5.4 (2 – 207)*
Hepatitis C virus antibodies	
Positive	43 (82.7%)
Negative	9 (17.3%)
Child Pugh score	9 (5 – 13)*
Child Pugh class	
A	9 (17.3%)
B	25 (48.1%)
C	18 (34.6%)
Model for end stage liver disease score (MELD score)	15 (6 – 31)*

*UCSF* University of California San Francesco

*Data is presented as median (range)

### Operative data

The operative data of the study cases were summarized in Table 2. Recipient PV was normal in 43 recipients (82.7%), attenuated in 7 recipients (13.5%), and partially thrombosed in 2 recipients (3.8%) requiring eversion thrombectomy.

**Table 2:** Operative data of the study cases

Variables	Number of patients (*N* = 52)
Ascites	
Yes	31 (59.6%)
No	21 (40.4%)
Recipient portal vein status	
Normal	43 (82.7%)
Attenuated	7 (13.5%)
Partial thrombosis	2 (3.8%)
Recipient main portal vein diameter (mm)	13 (6 – 22)
Graft weight (g)	968 (610 – 1521)*
Actual GRWR	1.22 (0.77 – 2.24)*
Graft hepatic veins number	
Single	34 (65.4%)
Multiple	18 (34.6s%)
Two	15 (28.8%)
Three	3 (5.8%)
Inferior right hepatic vein	6 (11.5%)
Segment VIII vein	5 (5.8%)
Segment V vein	9 (17.3%)
Eversion thrombectomy	2 (3.8%)
Donor portal vein variant	
Type 2	7 (13.5%)
Type 3	27 (51.9%)
Type 4	18 (34.6%)
Portal vein anastomosis number	
Single	9 (17.3%)
Double	43 (82.7%)
Portal vein reconstruction method	
Y graft in bench surgery	11 (21.2%)
Venoplasty of double portal vein stomas	9 (17.3%)
In situ double portal vein anastomoses	32 (61.5%)
Portal vein anastomosis revision	6 (11.5%)
Hepatic artery anastomosis number	
Single	52 (98.1%)
Double	1 (1.9%)
Hepatic artery anastomosis revision	5 (9.6%)
Hepatic artery difficulties	
No	43 (82.7%)
Recipient hepatic artery intimal dissection	5 (9.6%)
Size mismatch	2 (3.8%)
Double anastomoses	1 (1.9%)
Use of splenic artery	1 (1.9%)
Graft bile ducts number	
One	7 (13.5%)
Two	38 (73.1%)
Three	7 (13.5%)
Biliary reconstruction method	
Duct to duct	51 (98.1%)
Hepaticojejunostomy	1 (1.9%)
Number of biliary anastomoses	
Single	13 (25%)
Double	37 (71.2%)
Triple	2 (3.8%)
Operation time (min)	627 (435 – 930)*
Anhepatic phase duration (min)	45 (20 – 112)*
Cold ischemia time (min)	35 (15 – 132)*
Warm ischemia time (min)	45 (21 – 97)*
Blood loss (liters)	4.3 (1 – 11.5)*
RBCs units (units)	4 (0 – 30)*
Platelets (units)	1 (0 – 15)*
Fresh frozen plasma (units)	3 (0 – 29)*
Cell saver use	22 (42.3%)*

*GRWR* graft to recipient weight ratio, *RBCs* red blood cells

*Data is presented as median (range)

Type II PV was found in 7 cases (13.5%), which were reconstructed by direct venoplasty of the double PV stomas and single PV anastomosis in the recipient. Type III PV was found in 27 cases (51.9%). They were reconstructed by direct venoplasty of the double PV stomas and single PV anastomosis in the recipient in 2 cases (3.8%), Y graft interposition (from recipient PV) in the bench surgery and single PV anastomosis in the recipient in 2 cases (3.8%), and in situ double PV anastomoses in the recipient in 23 cases (44.2%). Type IV PV was found in 18 cases (34.6%) and was reconstructed by Y graft interposition (from recipient PV) in the bench surgery and single PV anastomosis in the recipient in 9 cases (17.3%), and in situ double PV anastomoses in the recipient in 9 cases (17.3%).

### Postoperative data

The postoperative data of the study cases were summarized in Table 3. Hepatic artery thrombosis occurred in 1 recipient (1.9%) that required surgical revision. Early right posterior PV stenosis occurred in 2 recipients (3.8%), one (1.9%) was managed conservatively and the other (1.9%) required radiology-guided dilatation and stenting. Early PV thrombosis occurred in 3 recipients (5.8%). One recipient (1.9%) was just segmental thrombosis and was managed conservatively, the other one (1.9%) required radiology-guided dilatation and stenting, and the last one (1.9%) required early surgical revision.

**Table 3:** Postoperative data of the study cases

Variables	Number of patients (*N* = 52)
Hospital stay (days)	21 (1 – 135)*
Intensive care unit stay (days)	6 (1 – 60)*
Morbidity	32 (61.5%)
Clavien-Dindo grade	
I	2 (3.8%)
II	8 (15.4%)
III-a	5 (9.6%)
III-b	9 (17.3%)
V	7 (13.5%)
Ischemia reperfusion injury^#^	2 (3.8%)
Primary non-function of the graft	1 (1.9%)
Acute cellular rejection	4 (7.7%)
Pulmonary complications	2 (3.8%)
Renal complications	2 (3.8%)
Gastrointestinal tract complications	
Diarrhea	1 (1.9%)
Persistent ascites	1 (1.9%)
Neurological complications	2 (3.8%)
Wound infection	1 (1.9%)
Management	
Bed side management	1 (1.9%)
Internal hemorrhage	3 (5.8%)
Collection	1 (1.9%)
Management	
US guided tube drainage	1 (1.9%)
Biliary complications	10 (19.2%)
Bile leakage	4 (7.7%)
Management	
US guided tube drainage	1 (1.9%)
ERCP	2 (3.8%)
Surgery	1 (1.9%)
Biloma	6 (11.5%)
Management	
US guided tube drainage	6 (11.5%)
Liver abscess	1 (1.9%)
Management	
US guided tube drainage	1 (1.9%)
Vascular complications	
Hepatic artery thrombosis	1 (1.9%)
Management	
Surgical revision	1 (1.9%)
Portal vein stenosis (right posterior vein)	2 (3.8%)
Management	
Conservative	1 (1.9%)
Stenting	1 (1.9%)
Portal vein thrombosis	3 (5.8%)
Extent:	
Right posterior portal vein	2 (3.8%)
Segment 7 portal branch	1 (1.9%)
Management	
Conservative	1 (1.9%)
Stenting	1 (1.9%)
Surgical revision	1 (1.9%)
Early surgery	10 (19.2%)
Early surgery cause	
Lavage for bile leak	1 (1.9%)
Portal vein revision	1 (1.9%)
Pack removal and hepatic artery revision	1 (1.9%)
Repair of intestinal injury	1 (1.9%)
Internal hemorrhage	3 (5.8%)
Pack removal	3 (5.8%)
Early mortality	7 (13.5%)
Early mortality cause	
Excess transfusion—ARDS	3 (5.8%)
Sepsis from bile leakage	1 (1.9%)
Portal vein thrombosis	1 (1.9%)
Ischemia reperfusion injury, kidney dysfunction	1 (1.9%)
Primary non-function	1 (1.9%)

*US* ultrasound, *ERCP* endoscopic retrograde cholangio-pancreatography, *ARDS* acute respiratory distress syndrome

*Data is presented as median (range)

^#^Defined as abnormal elevation of the liver functions in the early postoperative period, together with pathological examination of radiology guided liver biopsy

### Long-term outcomes

**Fig. 5:**
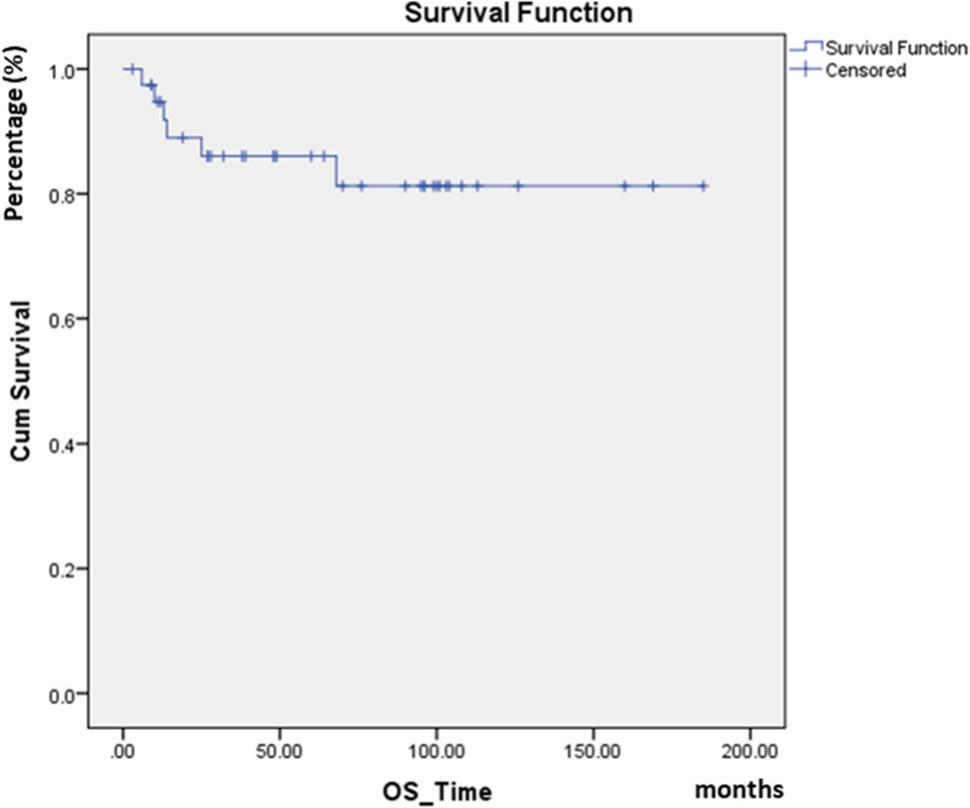
Kaplan–Meier overall survival curve of all study cases

The long-term follow-up data of the study cases were summarized in Table 4. The median follow-up duration was 54.5 months (4 – 185). The 1-, 3-, 5-, and 10-year survival rates were 91.9%, 86%, 81.2%, and 81.2%, respectively (Fig. 5). Late morbidities occurred in 14 recipients (26.9%). Biliary stricture occurred in 5 recipients (9.6%), 4 recipients were managed with endoscopic dilatation and stenting, while one recipient required surgical intervention. Late PV stenosis occurred in 2 recipients (3.8%) and was managed conservatively with close follow-up monitoring. Late mortality occurred in 6 recipients (11.5%), and the most common cause of late mortality was chronic rejection (2 recipients—3.8%).

**Table 4:** Long-term outcomes of the study cases

Variables	Number of patients (*N* = 52)
Late morbidities	14 (26.9%)
Late acute cellular rejection	2 (3.8%)
Chronic rejection	4 (7.7%)
Late biliary stricture	5 (9.6%)
Management	
ERCP	4 (7.7%)
Hepatico-jejunostomy	1 (1.9%)
Portal vein stenosis	2 (3.8%)
Management	
Conservative	2 (3.8%)
Incisional hernia	2 (3.8%)
Denovo tumors	1 (1.9%)
Type	
Ampullary tumor	1 (1.9%)
Management	
Surgical resection	1 (1.9%)
Late mortality	6 (11.5%)
Causes	
Chronic rejection	2 (3.8%%)
Biliary sepsis	1 (1.9%)
Fibrosing cholestatic hepatitis	1 (1.9%)
Cholestatic liver dysfunction	1 (1.9%)
Acute myocardial infarction	1 (1.9%)
Survival time (months)	54.5 (4 – 185)
Survival rate	
1 year	91.9%
years	86%
5 years	81.2%
10 years	81.2%

*ERCP* endoscopic retrograde cholangio-pancreatography

## Discussion

The global demands for liver transplantation have been progressively increasing worldwide in the last decades. On the other hand, there is a global shortage of organs that can meet the current demands resulting in a major organ shortage problem [20]. The problem is much exaggerated in countries where DDLT is lacking. In Egypt, DDLT program is still awaited because of several cultural and ethical aspects, making LDLT the only hope for patients with end stage liver disease (ESLD) with or without HCC [2, 3].

The availability of an appropriate living donor is the most vital step for LDLT. However, several factors can lead to rejection of the available living donor [8]. Transplant teams should exert all efforts to overcome potential factors that may lead to donor exclusion especially potential graft anatomical variations.

Portal venous reconstruction is a crucial and technically demanding step in liver transplantation [21]. Recent advancement of the imaging techniques of the liver enables the transplant surgeons to identify the intrahepatic anatomy precisely [5]. Anatomical variations of the right PV (RPV) are commonly encountered among potential donors for adult LDLT. The incidence of portal venous variations had been heterogenous between different series ranging between 10% and 35% [5, 8, 9]. In the current study, the incidence of RPV anatomical variations was 5.8% among right hemi-liver donors.

In the past, the anatomical variations of the RPV were considered contraindications for living donation. However, the improvement of surgical techniques and familiarity of the surgical procedure allowed transplantation teams to accept living donors with RPV anatomical variations allowing further expansion of the potential donor pool. Different surgical techniques had been described to overcome the RPV anatomical variations like back wall venoplasty (unification venoplasty), autologous Y-graft interposition utilizing either the recipient’s own PV confluence or cryopreserved iliac veins, in situ direct dual PV anastomoses, and Malatya approach utilizing autologous saphenous vein graft [10–13]. However, the most ideal surgical technique for reconstruction of RPV anatomical variations remains controversial.

In the current study we evaluated our center experience of different reconstruction techniques utilized in portal flow reconstruction for the right hemi-liver graft with PV anatomical variations. In the beginning of our experience, we utilized autologous Y-graft interposition technique utilizing the recipient’s own PV confluence for reconstruction of widely separated two RPV stomas. Autologous Y-graft interposition technique was used in 2 cases (3.8%) of Type III PV and 9 cases (17.3%) of Type IV PV in our series. It should be noted that our group experienced several complications after utilizing autologous Y-graft interposition technique especially thrombosis of right posterior PV (3 cases—5.8%). This is commonly attributed to twisting or kinking of the long right posterior PV [12, 22, 23]. Right posterior PV thrombosis could be managed with interventional radiology and stenting; however, one case (1.9%) required surgical exploration and revision of the right posterior PV anastomosis, but the patient outcome was poor. Similarly, Lee et al. evaluated the use of autologous Y-graft interposition technique in adult LDLT, and they found a high incidence of PV complications requiring PV stenting in 5 recipients (6.3%). They concluded that autologous Y-graft interposition technique is a technically demanding technique regarding the length, orientation, twisting, and buckling deformity of the autologous graft. It is associated with high incidence of functional PV stenosis requiring radiological interventions [24]. Yaprak et al. reported three main drawbacks of autologous Y graft interposition technique including that native PV has the risk of size and angle mismatch between the donors segmental PV branches and the vascular graft. Secondly, autologous native PV Y graft is associated with additional risks such as partial thrombosis of the vascular graft, increased wall thickness of the PV secondary to cirrhosis, and challenging dissection due to previous surgical and interventional procedures in the native liver. Finally, preexisting HCC in the native liver may threaten the vascular graft [8].

In the current practice, our group adopted the use of in situ double PV anastomoses in the recipient instead of autologous Y graft interposition technique for cases with widely separated two RPV stomas. In situ double PV anastomoses technique was used in 23 cases (44.2%) of Type III PV and 9 cases (17.3%) of Type IV PV in our series with adequate intraoperative PV flow. It should be noted that we did not experience any PV complications after adoption of in situ double PV anastomoses technique by our group. Kuriyama et al. recently evaluated the feasibility and outcomes of direct dual PV anastomoses in LDLT using the right hemi-liver graft with anatomical PV variations. They utilized direct dual PV anastomoses technique in 9 adult recipients with Type III PV variation and achieved successful results of all recipients [25].

In situ double PV anastomoses technique is simple and can be easily performed. Although it may be associated with some twist of the axis of main PV, however, it is not associated with significant PV flow changes. In few recipients with type III or IV PV, the two separate graft RPV stomas were anastomosed each to the corresponding recipient right anterior and posterior PV branches with closure of the recipient left PV stump to prevent over twisting of the axis of main PV. The main drawbacks of this technique are the prolonged warm ischemic time during performance of the dual PV anastomoses, and it could not be performed for recipients with higher PV thrombosis grades [25].

An additional important issue among potential donors with RPV anatomical variations is the association with right hepatic duct variations [26, 27]. Such association leads to an additional technical challenge for biliary reconstruction and increases the complexity of recipient operation. Kuriyama et al. found that biliary variations were found in 40% of donors with RPV anatomical variations [25]. Similarly, Kishi et al. found that biliary variations were more frequently found among donors with RPV anatomical variations (57%) when compared to donors with regular RPV anatomy (28.4%) [28]. In the current study, right hepatic duct variations were found in 45 cases (86.5%). Thirty-eight cases (73.1%) required multiple biliary anastomoses. Our group reported the importance of the use of intraoperative cholangiogram during living-donor right hemi-hepatectomy for evaluation of the donors’ biliary anatomical variations and defining the safe plane for right hepatic duct(s) division to decrease the incidence of biliary complications [29]. We experienced early biliary complications in 10 cases (19.2%) and late biliary complications in 5 cases (9.6%) in the current series. All of these complications were successfully managed by means of radiology guided drainage, endoscopic intervention, and surgical intervention if required [30].

The current study had some limitations. Firstly, it is a single center retrospective study which is liable to selection bias. Secondly, the number of included cases is relatively small owing to the unique nature of such vascular variation; however, a future multi-center study may help to recruit more cases.

## Conclusion

In conclusion, anatomical variations of the RPV are commonly encountered among potential living donors. Transplant surgeons should be familiar with such anatomical variations and different techniques for their reconstruction. We found that direct venoplasty of the double PV stomas and in situ dual PV anastomoses techniques are safe, feasible, and associated with successful outcomes. Utilization of potential living donors with RPV anatomic variations may help to expand the donor pool for LDLT.

## Data Availability

The study data will be available for review upon a reasonable request.
