# Correction to: Feasibility and outcomes of living‑donor liver transplantation utilizing the right hemi‑liver graft with portal vein anatomical variations

**DOI:** 10.1007/s00423-024-03226-x

**Published:** 2024-01-10

**Authors:** Ahmed Shehta, Mohamed Elshobari, Tarek Salah, Ahmad M. Sultan, Amr Yasen, Usama Shiha, Mohamed El-Saadany, Ahmed Monier, Rami Said, Mohamed S. Habl, Reham Adly, Basma Abd Elmoaem El Ged, Rasha Karam, Reem Khaled, Hassan Magdy Abd El Razek, Ehab E. Abdel-Khalek, Mohamed Abdel Wahab

**Affiliations:** 1 Gastrointestinal Surgery Center, Department of Surgery, Faculty of Medicine, Mansoura University, Gehan Street, Mansoura, Postal code: 35516 Egypt; 2 Liver Transplantation Unit, Gastrointestinal Surgery Center, Department of Anesthesia, Faculty of Medicine, Mansoura University, Mansoura, Egypt; 3 Liver Transplantation Unit, Gastrointestinal Surgery Center, Department of Radiology, Faculty of Medicine, Mansoura University, Mansoura, Egypt; 4 Liver Transplantation Unit, Gastrointestinal Surgery Center, Department of Hepatology, Faculty of Medicine, Mansoura University, Mansoura, Egypt

**Correction to: Langenbeck's Archives of Surgery (2023) 408:387**

10.1007/s00423-023-03115-9

Because of unclarity surrounding authorship, Ahmed M Elsabbagh was temporarily wrongly included in the author group of this paper. He has now been removed.

The original article has been corrected.

